# Testing the Differential Impact of an Internet-Based Mental Health Intervention on Outcomes of Well-being and Psychological Distress During COVID-19: Uncontrolled Intervention Study

**DOI:** 10.2196/28044

**Published:** 2021-09-15

**Authors:** Joep van Agteren, Kathina Ali, Daniel B Fassnacht, Matthew Iasiello, Gareth Furber, Alexis Howard, Lydia Woodyatt, Michael Musker, Mike Kyrios

**Affiliations:** 1 Wellbeing and Resilience Centre South Australian Health and Medical Research Institute Adelaide Australia; 2 Órama Institute for Mental Health and Wellbeing Flinders University Adelaide Australia; 3 College of Education, Psychology and Social Work Flinders University Adelaide Australia; 4 College of Nursing and Health Sciences Flinders University Adelaide Australia; 5 Health Counselling & Disability Services Flinders University Adelaide Australia; 6 Adelaide Nursing School Adelaide University Adelaide Australia

**Keywords:** COVID-19, internet-based interventions, mental health, well-being, intervention, study, impact, internet, online intervention, distress, resilience, depression, anxiety, stress

## Abstract

**Background:**

During COVID-19, the psychological distress and well-being of the general population has been precarious, increasing the need to determine the impact of complementary internet-based psychological interventions on both positive mental health as well as distress states. Psychological distress and mental well-being represent distinct dimensions of our mental health, and congruent changes in outcomes of distress and well-being do not necessarily co-occur within individuals. When testing intervention impact, it is therefore important to assess change in both outcomes at the individual level, rather than solely testing group differences in average scores at the group level.

**Objective:**

This study set out to investigate the differential impact of an internet-based group mental health intervention on outcomes of positive mental health (ie, well-being, life satisfaction, resilience) and indicators of psychological distress (ie, depression, anxiety, stress).

**Methods:**

A 5-week mental health intervention was delivered to 89 participants using the Zoom platform during 2020. Impact on outcomes of distress, well-being, and resilience was assessed at the start and end of the program with multiple analysis of variance (MANOVA) and reliable change indices (RCIs) being used to determine program impact at the group and individual levels, respectively.

**Results:**

The intervention significantly improved all mental health outcomes measured, (*F*_6,83_=5.60, *P*<.001; Wilks Λ=.71; partial η^2^=.29) showing small to moderate effect sizes on individual outcomes. The largest effect sizes were observed for life satisfaction and overall well-being (η^2^=.22 and η^2^=.2, respectively). Larger effect sizes were noted for those with problematic mental health scores at baseline. A total of 92% (82/89) of participants demonstrated reliable change in at least one mental health outcome. Differential response patterns using RCI revealed that more than one-half of the participants showed improvement in both mental well-being and psychological distress, over one-quarter in outcomes of well-being only, and almost one-fifth in distress only.

**Conclusions:**

The results provide evidence for the significant impact of an internet-based mental health intervention during COVID-19 and indicate the importance of assessing dimensions of both well-being and distress when determining mental health intervention effectiveness.

## Introduction

### Mental Well-being and Psychological Distress as Dual Dimensions of Mental Health

A commonly cited definition of mental health is the one postulated by the World Health Organization: “a state of well-being in which the individual realizes his or her own abilities, can cope with the normal stresses of life, can work productively and fruitfully, and is able to make a contribution to his or her community” [1]. Despite this definition — and numerous other readily used mental health definitions — incorporating positive facets of mental health, mental health care, and the research that underpins it is overwhelmingly focused on understanding and solving problems related to mental disorders [2]. The same applies for mental health intervention research, where historically, efforts have centered around treating or intervening in mental disorders, invariably focusing on “mental health conditions” or “mental illnesses” that significantly affect cognition, emotion, and behavior that may lead to dysfunction or disability [3,4]. Concurrently, research into mental health has focused on the related outcome of psychological distress [5], a state of emotional suffering that is typically characterized by symptoms of depression and anxiety [6].

Although a predominant emphasis on addressing psychological distress and mental disorder still persists, the COVID-19 pandemic has made the need to focus beyond mental disorders in clinical populations more salient. The far-reaching societal consequences of the pandemic increased the need to investigate how the general population can maintain positive and adaptive states of mental health and how these positive and adaptive states can be utilized to buffer against developing more complex problems [7,8]. Although the pandemic brought promotion of positive mental health, or alternatively, states of “mental well-being,” more front-of-mind, these outcomes have for decades already been advocated to be an important standalone mental health outcome and therapeutic avenue, in contrast to the “traditional” focus on merely reducing pathology [9,10]. Mental well-being specifically refers to a state where people generally feel good (ie, experience more positive than negative emotions, feel a sense of life satisfaction) and feel that they can function fully (eg, are able to self-actualize, self-realize, and have a sense of meaning) [11]. In other words, they perceive enjoyment and fulfilment with one’s life as a whole [12]. Decades of research by well-being pioneers such as Diener [13], Ryff [14], and Keyes [15], followed by researchers operating in the field of positive psychology [16], broached the important role of promoting mental well-being in the general population. A now large and established body of research clearly links the presence of mental well-being to a range of desirable physical health (eg, longevity and healthy aging, reduced hospital use) and mental health (eg, lower rates of suicide ideation, more healthy coping) outcomes [17-23].

Psychological distress and mental well-being do not merely occupy opposite ends of a single continuum, and further research is required to understand the complex relationship between the constructs, particularly in intervention research [24,25]. For example, high well-being indicators do not simply equate to being mentally healthy, as both well-being *and* the absence of distress or disorder are required to be mentally healthy [24,26]. Similarly, mental well-being is not simply the opposite of mental disorder, but rather is an outcome that can co-occur and be juxtaposed to the presence of mental disorder and psychological distress. A scoping review conducted in 2020 identified over 80 scientific publications providing supportive evidence that well-being and disorder/distress are considered to be negatively related but independent outcomes, with both aspects associated with good mental health [27]. First, empirical studies have shown the importance of maintaining and improving the mental state of well-being for the *prevention* of potential mental disorder [28-30]. Second, studies have shown that improving states of mental well-being in people with current mental disorders can impact rates of recovery [31,32].

### The Need for Scalable Interventions Designed to Address Both Well-being and Distress

While definitive research on the long-term impact of COVID-19 on mental health outcomes across the general population is heterogeneous and not clearly established, with more research needed to determine which population groups are most affected, academic research published since the outbreak of the pandemic generally points to a negative immediate consequence [33-39]. These adverse mental health consequences are often the result of societal restrictions and policies, rather than infection with (or fear thereof) the virus itself [40-42]. This is particularly the case for countries where COVID-19 caseloads have been comparatively low such as Australia. As of July 10, 2021, Australia had a total of 31,017 confirmed cases of COVID-19, resulting in 910 deaths (2.93% death rate) [43]. This death rate, 3.6 per 100,000, is very low compared with other countries such as the United States (184.9 per 100,000) and the United Kingdom (192.5 per 100,000) [44]. Despite this attenuated impact, various studies point to an immediate impact on outcomes of well-being and distress in Australia, particularly in at-risk population groups [45-48]. For instance, Batterham et al [49] found that participants in Australia who had financial distress, social impairment, and work impairment were most impacted. Similarly, Li et al [50] found that young people, who due to their age are already at higher risk of mental health problems, showed elevated mental health problems. University students, who generally display high rates of mental health problems and lower well-being [51], similarly demonstrated mental health problems immediately after the pandemic began [52].

As a result of the observed and anticipated consequences, there have been widespread calls to proactively intervene in mental health by targeting distress and the well-being of the general population [8]. The importance of addressing states of well-being and distress conjointly is advocated by proponents of dual-factor models [24,53], well-being therapy [54], the recovery movement [10], positive (clinical) psychology [2], and positive psychiatry [55]. These distinct streams in mental health care propose that, by taking both well-being and distress into account, the way we deliver mental health care across the spectrum of mental health, ranging from self-help options to treatment of recurrent lifetime disorders, can be improved. For example, mental health interventions incorporating a focus on well-being have the potential to prevent more severe mental disorder, can augment treatment, or can be used as early intervention for subclinical issues. In other words, such interventions can be used as a target to promote mental health in the general population using psychological interventions, addressing prodromal symptoms [26].

A range of behavioral and psychological interventions exist that have proven to positively impact mental well-being in nonclinical settings [56-58]. The most renowned of these psychological interventions stems from the work of positive psychology [59], but various distinct psychological interventions will have a significant impact, depending on a range of moderators including target population and delivery format [58]. One viable delivery format that has been shown to be able to have a sustainable impact in the general population is the use of internet-based solutions, which can be deployed at a larger scale and can be used irrespective of the presence of physical restrictions (eg, lockdowns) [60,61]. This is particularly the case for mental health interventions that target lower-intensity problems or issues related to well-being and mental health promotion, where support by mental health professionals is less warranted [62]. A rapid review by Rauschenberg et al [63] found good evidence for the short-term impact of online mental health interventions during COVID-19 for mental disorder, with evidence for mental health promotion interventions still being sparse.

A by-product of utilizing internet-based interventions with a stronger focus on well-being is that it can also help improve the current gap in service delivery for those experiencing symptoms of mental disorder, as it may help to address various challenges of the mental health system in clinical populations, including access issues, stigma, or “treatment resistance” to name a few [64,65]. For instance, providing complementary or integrated well-being intervention programs may reach clients and community members who do not respond to traditional treatment or do not access these due to associated stigma [66]. Adopting a strong emphasis on well-being also diminishes the reliance on using outcomes of distress and illness as the only indicators of treatment effectiveness. For instance, a poor response to psychological interventions is not uncommon, with nonresponder estimates of 30%-40% being documented [67]. Nonresponse in outcomes of distress is often seen as a lack of treatment impact, particularly in treatment models where the main focus is elimination of symptoms. As mental well-being is related to both prevention of mental disorder and recovery from illness, it plays a fundamental role in personal and functional recovery, which may be considered to be a proxy for future treatment impact [18,27].

### The Need to Look Beyond Group Averages

In order to determine the merit of psychological interventions on improving mental health outcomes, scholars increasingly stress the importance of measuring both states of distress and well-being to best evaluate mental health intervention impact, a recommendation that comes with an often-overlooked nuance [68]. The impact of an intervention is often determined by comparing an average shift in scores of a cohort of participants using specific outcome measures [69]. For instance, an intervention targeting distress is thought to be beneficial if it can demonstrate an average significant and meaningful change in average scores [70] using, for example, a validated depression outcome measure such as the Depression Anxiety and Stress Scale (DASS-21) [71]. While average improvements are an important way to assess the potential impact of interventions on *groups*, it does not indicate whether interventions are efficacious or even suitable on an individual level [72].

Comparison of average changes furthermore obscure any possible *differential* impact of interventions on outcomes of mental well-being or distress within individuals [73]. Average improvements in mental well-being and mental disorder outcome measures at a group level do not necessarily mean that each individual demonstrates equal changes in both measures after receiving an intervention. These types of analyses leave intra- and even interindividual responses unclear and limited in their ability to detect how different individuals may respond in relation to different mental health outcomes. Thus, these generic approaches obscure important results that provide information as to what interventions work, on what dimensions they apply, and who specifically benefits from them. Subsequently, these studies provide limited guidance for practitioners and other stakeholders who wish to understand the nuances of how different interventions affect outcomes within (as opposed to between) individuals and then applying this knowledge to the way they provide mental health care [73].

Scientific studies investigating mental health interventions have not readily reported analyses of intraindividual change across both dimensions of mental health. A notable recent exception is a study by Trompetter et al [68], who investigated the impact of a self-help Acceptance and Commitment Therapy (ACT) intervention for people with clinical depression or anxiety. The authors utilized reliable change indices (RCIs) [74,75] to determine whether individuals demonstrated a meaningful change in their mental health outcomes. The researchers found that improvements in mental health or mental disorder did not necessarily co-occur. While 31% of participants improved across both dimensions, only 12% improved in aspects of positive mental health, and 57% improved in symptoms of distress. Additionally, results further differed for participants with depression and anxiety, showing how outcomes of well-being can interact differently with different outcomes of illness. Thus, analysis of the results using RCI not just enabled assessment of the overall effectiveness of the intervention for these 2 clinical populations but also gave better insight about for whom the intervention showed the most effect.

Additionally, approaches such as those taken by Trompetter et al [68] enable the assessment of change while taking baseline scores into consideration. Baseline scores have a clear impact on the summary of change that is potentially possible for each individual [76]. Positive reliable change cannot be expected if participants start at a baseline level that is not conducive to further improvement. Studies that focus on a reduction in pathology, both mental and physical, therefore typically use severity classifications to inform participant criteria for potential participant recruitment. For example, researchers might only include participants who meet the mild depression cutoff on measures such as the DASS-21 or Hamilton Anxiety and Depression Scales to ensure that participants actually show signs of current distress [77,78]. In contrast, studies that investigate the impact of interventions on mental well-being typically do not consider cutoffs as an important starting factor, neglecting the critical impact of baseline scores; this is an important relation to assess considering the precarious situation of mental health and mental health care and the calls for mental health promotion interventions for the general population during the COVID-19 pandemic [8,48,62].

This study aimed to advance the literature in a number of ways. First, it aimed to determine the impact of an internet-based psychological intervention on mental health outcomes during COVID-19 in Australia. It aimed to add to the currently lacking evidence [63] on the benefit of (internet-based) interventions to promote mental health and well-being, as opposed to targeting clinical symptoms in clinical populations. Second, it aimed to investigate whether the findings reported by Trompetter et al [68] can be replicated in a nonclinical, general population sample; the study aimed to determine differences in reliable change in outcomes of both mental well-being and psychological distress. Rather than using an intervention based on a clinical treatment approach (ACT in the case of the study by Trompetter et al [68]), our study aimed to show this differential impact of a universal, group-based, mental health intervention designed to promote mental health in the general nonclinical population. Finally, the study aimed to examine the effect of baseline differences in well-being and psychological distress on the effectiveness of the intervention.

## Methods

### Study Design

This study was designed to establish whether a range of mental health outcomes would increase due to exposure to an internet-based psychological intervention and to test whether individuals would display improvements differentially across these outcomes using reliable change analysis. The study was an uncontrolled intervention study comparing data collected using an internet-based measurement tool at the beginning of the intervention compared to data by the end of the intervention. The study was approved by the Flinders University Human Research Ethics Committee (PN 2163).

### Participants

Participants for this study were from 2 cohorts. The first consisted of adults (18 years or older) from the general population, while the second cohort consisted of adult university students. Participants from the general population signed up via an online website, which was promoted via local print media and radio, an email newsletter for the South Australian Health and Medical Research Institute (SAHMRI), and social media posts on Facebook and Twitter. The second cohort consisted of students who were recruited through one of the major universities in South Australia. No specific inclusion or exclusion criteria applied other than the requirement to be adult, understand the English language, and have access to an internet-enabled device with Zoom [79]. There was no face-to-face contact with any of the participants.

### Recruitment

Recruitment was conducted over 6 months from March 2020 until July 2020. Recruitment procedures for the general population and the student population differed slightly. At the university, the study was advertised as a tailored, free, online program to improve mental health and well-being developed by SAHMRI and Flinders University. Promotional information for students was distributed via university emails, student associations, and social media including details about the program, facilitators, and the delivery format. Recruitment material highlighted the importance of well-being and mental health, and importantly, this period coincided with the ongoing impact of COVID-19. Recruitment material provided information about the development and individual components of the program. Interested participants provided their email address, name, and college and were sent an invitation to complete a measurement about their mental health and well-being 1 week before starting the first online session. Measurement was to be completed prior to commencing the intervention. Participants were not obliged to participate in the research study in order to attend the individual sessions. Within the general population, participants could enroll via a website with detailed information about the program or were recruited via partner organizations who were interested in promoting a well-being program to their staff.

Participants were sent an invitation to complete a measurement about their mental health and well-being before starting the initial online session. All participants were invited to attend 5 2-hour weekly sessions hosted online. While initially the study was planned to be conducted in person, COVID-19 restrictions in Australia required online delivery of the program via Zoom [79]. By the final session, participants were invited to complete a second measurement, which was used to determine the post-intervention score outcomes. This was emailed to participants 1 week before the final session, to be completed prior to attending the final session so that participants could track any changes observed since the original measure.

### Intervention

The “Be Well Plan” is a 5-week, group-based psychological program that helps participants create a personalized mental health and well-being plan by experimenting with a variety of resources and evidence-based activities to improve mental health and well-being. The program can be delivered in person in small- to medium-sized group settings (ranging between 10 and 40 participants) or alternatively online via platforms such as Zoom [79], the format utilized for this study.

Program facilitators for the training do not need to be trained mental health professionals, but rather are upskilled to be able to facilitate the training in an engaging and safe way, thereby improving the scalability of the program without further constraining existing health care resourcing. The trained trainers are required to participate in a minimum of 26 hours of face-to-face training. For this study, 6 trained trainers were involved in the delivery of the intervention with a variety of professional backgrounds including well-being research, counselling, workplace development training, and clinical psychology. Facilitators were either staff involved in the development in the intervention (n=4) or colleagues who have a professional training background and were involved in early testing rounds of the intervention (n=2).

The program was designed using a rigorous intervention development process called intervention mapping and among other techniques, relied on the use of co-design with end users and stakeholders [80]. The program aims to impact both outcomes of well-being and outcomes of distress by incorporating evidence-based activities aimed at promoting mental health. Included activities were derived from a large systematic review on distinct psychological interventions aimed at improving outcomes of well-being conducted by members of the research team [58]. A particular strength is that the program is tailored to participants’ unique mental health needs and interests of individual participants. A detailed description of the program and its development will be published in a separate manuscript. A general overview of the intervention can be found in Table 1.

**Table 1:** Summary of the 5 sessions of the Be Well Plan.

Session	Description
Session 1: Getting on the same page	Participants explore reasons for participating in the program, including their personal drivers. They also acquire basic knowledge of mental health and its malleability. This aims to stimulate a mindset for change. They continue by exploring the evidence for different psychological interventions and start creating their first Be Well Plan. They do this by choosing one of many formats of practicing mindfulness and setting a goal on how to practice it during the week. They get introduced to the formation of tiny habits and implementation intentions as a technique to improve the chance of goal attainment.
Session 2: Using your mental health profile	Participants reflect on session 1. They are introduced to the concept of self-compassion (as opposed to self-criticism) and how it can be used to learn from failure and shape our thinking patterns. They practice a self-compassion activity. They subsequently use their own measurement result stemming from the integrated assessment to focus on an outcome they want to work on (well-being, resilience, mood, anxiety, stress, health) and are introduced to activity finders: flow charts that map evidence-based activities to each of the activities. They pick one activity from the activity bank to add to their Be Well Plan and set new goals for the week. They will be introduced to the use of prompts and reminders as another method to increase goal attainment.
Session 3: Your resources and challenges	Participants reflect on week 2. They work with (and are reminded of) existing resources to their own mental health via 2 practical activities. In the first one, participants choose pictures that display sources of meaning in their life; in the second one, participants identify core values that can be used to guide goals. They then use a simple questionnaire to identify a key resource or challenge they want to work on. They are introduced to a second activity finder that maps evidence-based activities to various challenges and resources. They pick a new activity from the activity bank to add to their Be Well Plan. They finish the session by adjusting their Be Well Plan.
Session 4: Stress, coping, and resilience	Participants reflect on week 3. This session focuses on stressful times and effective ways to cope (avoidance-focused coping versus more helpful ways, such as problem-focused coping). They are then walked through various ways of coping using psychological techniques and theories, including identification of cognitive traps and the use of thought defusion. They are asked to identify social supporters for challenging times and are reminded of various professional services. They then choose 1 new activity specifically focusing on stress and resilience. They are actively asked to reach out to a social supporter as part of their weekly activities.
Session 5: Future-proofing your Be Well Plan	Participants reflect on the past 4 weeks. They are asked to complete a new measurement and investigate how their outcomes have changed over the 4 weeks. The facilitators introduce the concept of realistic optimism, growth, and the fact that progress comes with ups and downs. Participants work on practicing positive reframing as a way to deal with mistakes and setbacks. They then build their final Be Well Plan, which aims to summarize key learnings from the previous weeks into a standalone plan. They summarize what their best possible mental health looks like, highlight their unique drivers and motivators, and existing resources and challenges in their life. They set a longer-term goal and choose the activities they wish to add to their Be Well Plan. They identify their key supporters and reflect on what support services they need in case of emergency.

### Measures

Outcomes measured in this study included positive mental health and psychological distress, which are mental health outcomes that are most relevant to nonclinical populations.

#### Mental Well-being

Mental well-being was captured using the Mental Health Continuum Short-Form (MHC-SF) [81], which allows the calculation of a generic well-being score as well as subscores for emotional (hedonic), psychological (eudaimonic), and social well-being. The scale showed high internal reliability, with a Cronbach α of 0.94. Furthermore, the Satisfaction With Life Scale (SWLS) [82] was used to provide an alternative measure of general well-being, which similarly showed good internal reliability: α=0.89.

#### Psychological Distress

Psychological distress was captured with the DASS-21, which offers clear cutoff points for the level of severity of symptoms. This facilitates grouping of scores into “normal,” “mild,” “moderate,” “severe,” and “extremely severe” symptoms of psychological distress for the domains of depression (α=0.91), anxiety (α=0.82), and stress (α=0.86).

#### Resilience

An additional outcome of interest was resilience, or the perceived ability to withstand stress, a relevant outcome considering the impact of COVID-19 on stress levels. Resilience was assessed using the Brief Resilience Scale [83], which looks at whether respondents feel they are able to deal with stressful situations. This tool also comes with cutoffs for low, normal, and high resilience. Internal consistency was high: α=0.89.

### Statistical Analysis

#### Evaluating the Impact of the Intervention

Data analysis was conducted in RStudio and SPSS version 27. To determine the average change between baseline and the final session, a repeated measures multiple analysis of variance (MANOVA) was completed. A MANOVA was chosen in order to account for the considerable overlap between selected mental health outcome measures. Where needed, data were transformed to deal with the presence of nonnormality; as results did not change between analyses on transformed and untransformed data, the untransformed results were used in this article. One univariate outlier was excluded from the analysis as it significantly impacted the results. Multivariate outliers did not affect the results and were therefore left unchanged. Multicollinearity was assessed using bivariate correlations, revealing that the majority of outcomes showed a positive or negative correlation between .40 and .65. Partial eta squared was used as a measure of effect size, where 0.02 equals a small effect, 0.13 a medium effect, and 0.26 or higher a large effect [84].

#### Analysis of Within-Individual Changes Post-Intervention

Within-individual changes in outcomes were assessed by calculating an RCI using the traditional method for assessment of reliable change as suggested by Jacobson and Truax [75]. The RCI was calculated by subtracting an individual’s post-intervention score from their baseline score and subsequently dividing this difference score by the standard error of the difference for the measurements used. The standard error of the difference was calculated using the following formula:

SEdiff = SD_x_ * √(1-r_xx_),

where SD_x_ refers to the SD of the difference scores and R_xx_ refers to the correlation between scores on the pre and post measurements. Any change larger than 1.96 (2 SDs) was considered a reliable change.

#### Assessment of Baseline Differences in Intervention Outcomes

The impact of baseline well-being and psychological distress on outcome impact was assessed with independent samples *t* tests. Where distributions were nonparametric, both normal and parametric tests (ie, Mann-Whitney U-tests) were run. As the results for parametric and nonparametric tests returned similar results, results presented report the parametric results (ie, results for the *t* tests). Participants were grouped into “high” vs “low” well-being according to the cutoffs on the MHC-SF [81] as well as “high” vs “low” life satisfaction according to a cutoff on the SWLS [82]. Furthermore, participants were also grouped into “distressed” vs “no distress” if they met any of the cutoffs for “mild distress” on one of the DASS-21 subscales [71].

## Results

A total of 240 participants took part in the training, of which 140 participants provided consent to be studied. Of these 140, a total of 90 participants provided data at both timepoints. A total of 89 participants were included in the analysis, after excluding a severe outlier.

### Participant Characteristics

The average participant age was 38.67 (SD 13.06) years. A total of 59 participants (59/89, 66%) were female, who had an average age of 40.05 (SD 14.07) years. Of the participants, 19 (19/89, 21%) were male, with an average age of 37.37 (SD 10.43) years. “Prefer not to say” was answered by 11 participants (11/89, 12%). Most participants were employed (69/89, 77%; average age: 40.71, SD 13.26 years) and not studying (57/89, 64%; average age: 43.25, SD 12.40 years). When comparing students (32/89, 36%; average age: 30.53, SD 9.98 years) to participants from the general population, it was noted that mental health baseline values were significantly worse for students for all outcomes: *F*_6,82_=3.94, *P*=.002; Wilks Λ=.78; partial η^2^=.22.

### Evaluating the Impact of the Intervention

Comparison of pre- and post-intervention scores showed a significant change in mental health variables across time: *F*_6,83_=5.60, *P*<.001; Wilks Λ=.71; partial η^2^=.29. Table 2 displays the positive changes in all individual domains and the relevant test statistics, showing a significant positive change in all outcomes measured. Effect sizes for significant outcomes ranged between small and moderate, with the largest significant improvement noted in life satisfaction and the smallest improvements in anxiety.

**Table 2:** Pre- and postintervention scores for outcomes of mental well-being, psychological distress, and resilience in the total sample (n=89).

Outcomes	Preintervention		Postintervention					Statistics		
	Score, mean (SD)	Participants with problematic scores^a^, n (%)	Score, mean (SD)		Participants with problematic scores, n (%)	*F* (*df*)			*P*	*η* ^2^
Overall well-being	45.81 (11.18)	51 (57)	49.39 (12.19)		41 (46)	22.43 (1)			<.001	.20
Life satisfaction	22.01 (5.93)	26 (29)	24.46 (6.48)		21 (24)	25.29 (1)			<.001	.22
Distress due to mood	10.00 (8.91)	42 (47)	7.91 (7.37)		32 (36)	9.44 (1)			.003	.10
Distress due to anxiety	6.54 (6.77)	34 (38)	5.33 (5.71)		26 (29)	5.45 (1)			.02	.06
Distress due to stress	13.03 (8.18)	32 (36)	10.67 (7.94)		25 (28)	11.86 (1)			<.001	.12
Resilience	3.27 (0.76)	26 (29)	3.45 (0.75)		21 (24)	10.84 (1)			.001	.110

^a^Problematic scores refer to scores where participants did not meet the cut-off for high well-being, normal resilience, or no presence of distress.

### Analysis of Within-Individual Changes Post-Intervention

Analysis of reliable change indicated that a total of 92% (82/89) of the participants demonstrated improvement in at least one of the domains of the outcomes measured. Of these 83 participants, 51% (42/82) showed both improvements in well-being and indicators of distress, whereas 29% (24/82) only showed improvement in well-being and 20% (17/82) only showed improvements in distress. Further, response patterns differed for the various distress categories.

Those who met the threshold for mild depressive symptoms and displayed a reliable improvement largely showed reliable change in both well-being and distress (25/35, 71%), with additional proportions demonstrating improvement in either well-being (5/35, 14%) or distress (5/35, 14%). Those participants who met the threshold for anxiety demonstrated reliable improvement in both anxiety and well-being (15/29, 52%) or well-being alone (13/29, 45%); only one participant (1/29, 3%) demonstrated reliable change in anxiety scores only. The majority of those who met the threshold for stress showed reliable change in well-being outcomes only (19/31, 61%), with 7 participants (7/31, 23%) showing a reliable change in both distress and well-being and 5 participants (5/31, 16%) showing reliable change in distress only.

### Assessment of Baseline Differences in Intervention Outcomes

Change in mental health outcomes significantly differed for those with “low” baseline values compared with those with “high” baseline scores before the intervention. As expected, significant changes were found for life satisfaction and distress across all categories, with those reporting lower baseline scores experiencing significantly lower change in outcome scores (see Table 3). Despite students showing significantly worse baseline mental health problems, no significant interaction effect was noted (*F*_6,82_=1.70, *P*=.132; Wilks Λ=.89; partial η^2^=.11), showing that the change in the mental health outcome was not significantly different for students versus nonstudents.

**Table 3:** Comparison of the outcomes of mental well-being, psychological distress, and resilience in the total sample of participants between participants with and without problematic scores at baseline.

Outcomes	Participants with problematic scores^a^ at baseline				Participants with healthy scores at baseline			Statistics			
	Pre-intervention, mean (SD)	Post-intervention, mean (SD)	N	Pre-intervention, mean (SD)		Post-intervention, mean (SD)	N		*t* (*df*)	*P*	*d*
Well-being	38.18 (7.61)	42.47 (10.62)	51	56.05 (5.56)		58.68 (6.78)	38		1.02 (86)	.31	0.21
Life satisfaction	14.62 (3.20)	18.77 (6.56)	26	25.06 (3.65)		26.81 (4.80)	63		2.14 (87)	.04	0.50
Mood problems	17.76 (6.69)	13.05 (7.10)	42	3.06 (2.79)		3.32 (3.64)	47		3.79 (55)	<.001	0.84
Anxiety problems	13.35 (6.06)	8.53 (6.41)	34	2.33 (2.24)		3.35 (4.20)	55		6.23 (55)	<.001	1.46
Stress problems	22.06 (5.39)	16.44 (8.09)	32	7.96 (4.04)		7.44 (5.77)	57		3.37 (44)	.002	0.85
Resilience	2.29 (0.40)	2.69 (0.70)	25	3.66 (0.45)		3.74 (0.53)	64		2.86 (87)	.005	0.68

^a^Problematic scores refer to scores where participants did not meet the cut-off for high well-being, normal resilience, or no presence of distress.

## Discussion

This study demonstrated that an internet-based mental health program could elicit differential change in outcomes of mental well-being and psychological distress in a nonclinical population during COVID-19. Results demonstrated that mental health outcomes in the sample improved from the beginning to the end of the intervention and that participants with poorer baseline scores had a significantly better response compared to those with greater average baseline scores. Furthermore, the results highlighted that reliable change in outcomes of mental well-being and psychological distress could occur independently, with type of distress (depression, anxiety, stress) resulting in differential response patterns.

The results here bolster the evidence that internet-based interventions can play a significant role in dealing with the mental health consequences of the pandemic [60,61,85,86]. This intervention was delivered over internet-based teleconferencing software using trained professionals outside of a clinical setting. The training, which focuses on promoting mental health, not the specific treatment of mental illness, was designed to be able to be delivered without the reliance on clinical staff. This approach of upskilling nonclinical staff to deliver mental health training has successfully been utilized by our team before and facilitates scalability and reach of the solution [87,88]. Mental health systems are typically under-resourced, which has further deteriorated during COVID-19, fueling calls for innovative solutions as the one presented in this article, particularly those that safeguard ethical principles [85,89]. Finding positive results for interventions that aim to promote mental health in both outcomes of well-being and distress in a general population under duress makes for promising standalone first-line interventions or as solutions to deal with existing system issues (eg, waitlists) [18].

While the significant impact of the intervention in a general nonclinical population was promising, the use of the term “nonclinical” warrants attention. This term mainly reflects the inclusion criteria rather than an actual lack of clinical symptoms among participants. As may be expected, the majority of participants did in fact show mild symptoms of distress (53/89, 60%). This proportion, at first glance, may appear higher than typically reported in the general population, such as the frequently cited “one in five who are struggling with symptoms of a common mental disorder” [90]. This increased rate may partly be attributed to the result of the pandemic but may also be explained by the fact that our sample stemmed from 2 different population pools: the general adult population and tertiary university students. Previous studies have demonstrated that mental health outcomes in students are worse across many domains than in the general population [51,91,92], which was supported in our study. These findings first elicit the need to thoroughly investigate and improve the mental health of, demands on, and lifestyle of our tertiary students [93], but second, highlights an area that requires the attention of researchers who may use student cohorts for their mental health research and wish to compare their findings to a sample of the general population.

The findings support previous research indicating that improvements in distress do not automatically result in improvements in well-being and vice versa. Similar to the findings by Trompetter et al [68], this study showed that, while participation in the intervention led to overall improved mental well-being and reduced psychological distress, not every individual improved in both domains. These results provide an additional piece of evidence that supports the independence of outcomes for mental well-being and mental disorder [27]. As research by dual-factor model scholars such as Keyes [24] and Greenspoon and Saklofske [53] proposes, the ultimate end goal of our mental health care system ought to strive for “complete” states of mental health, that is states of high well-being and no distress or symptoms of mental disorder in as many people as possible. This indeed would be the expectation if we were to deliver mental health care that lived up to and met the contemporary definitions of mental health such as the one posited by the World Health Organization [1]. Results here show though, that in order to meet this aspirational international standard, it is critical to systematically measure both outcomes of well-being as well as psychological distress when assessing the impact of psychological interventions in research and practice [27].

The difference in intra-individual responses between psychological distress types furthermore points to the complex relationship between states of well-being and distress and their outcome measures [25]. Most participants who showed changes in depression had simultaneous improvements in well-being outcomes, which was not the case for anxiety or stress. A possible explanation for these patterns of change points to the inherent similarity between the construct of depression and happiness [29]. For instance, evidence for dual-factor models is less convincing for people with severe depression [94], and these models do not work as well when outcome measures specifically take advantage of the emotion of “happiness” or other affective states of hedonic well-being. In the data presented here, even the moderate correlation (r=.5) between the measure of life satisfaction (SWLS) and general well-being (MHC-SF), which captures the factors of hedonic, eudemonic, and social well-being, shows that these 2 well-being measures vary substantially. Therefore, it is essential to carefully consider the most appropriate measure when assessing the impact of interventions or treatment modalities, as this decision has consequences for the perceived impact of the intervention in both types of outcomes [95].

Our finding that effect sizes were higher for people meeting the threshold of psychological distress or low well-being is encouraging. In addition to providing insight into the impact of interventions for individuals with different states of mental health, promoting the importance of baseline mental health on interventions has practical implications for treatment models. There is an ever-increasing burden of mental health problems, with mental health systems around the globe feeling the strain [96,97]. Advocates of change have long been calling for new solutions to help support the provision of complementary services and group-based mental health interventions that can be delivered online and in person. The Be Well Plan program offers a solution that can be implemented to ameliorate current system pressures, complementing other accessible solutions such as low-intensity cognitive behavioral therapy [98,99]. Consequently, it is important to determine an effective model of universal programs that have the greatest impact on mental health outcomes, while reducing the burden of disease. While more research is needed to understand the particular effectiveness of each program component on different mental health outcomes [73], our findings support the need to improve and innovate lower tiers in evidence-based, stepped-care models or stimulate a stronger focus on well-being within integrated care models [100,101].

This research has limitations requiring comment. First, the results stem from an uncontrolled study, which means that the evidence is not conclusive in supporting the efficacy of the intervention. The intervention was delivered in response to the immediate mental health demands in the community during the pandemic; therefore, the team made a conscious call to deliver the intervention to anyone who signed on immediately, rather than randomize them into waitlists. This design limitation, however, does not impact the validity of the findings, as a core aim of the study was to explore a within-subject change design. That said, it is important to compare and verify reliable change between an active intervention and a comparable control condition in future studies. Uncontrolled studies, for example, do not account for various confounding factors (eg, the impact of extraneous events and lifestyle factors). In this case, the study was conducted while the COVID-19 pandemic was ongoing, which could have had a significant impact on mental health outcomes during this extraordinary period [45] and therefore would have impacted the results one way or the other.

Second, the results presented only refer to short-term outcomes, clearly warranting the need to examine long-term changes. For instance, improvement in well-being has been shown to be associated with long-term recovery of mental disorder in observational studies [31]. It is hypothesized that well-being may therefore be a therapeutic focus for long-term (symptom) recovery [18], but a rigorous body of research intervention studies is yet to be established [27].

Third, the current results apply to a general population cohort; extrapolation to clinical populations should be used with caution. Although the sample did include participants that showed higher distress levels, the presence of psychological symptoms does not equal the presence of disorder, which requires assessment using different outcome measures [5]. The results presented by Trompetter and colleagues [68], however, did apply to clinical populations and therefore pose a reference point for those working in the clinical area.

A fourth and similar limitation lies in the specificity of our outcome measures. This study used a general measure of distress implying that the results should not be generalized to determining the impact on explicit symptoms of mental disorder [102]. The current results only refer to the differential changes in well-being and distress, demonstrating that changes in outcomes of mental well-being and psychological distress do not automatically go hand in hand after participation in a mental health intervention. Both outcomes should be considered and assessed when investigating the impact of psychological interventions and changes in mental health outcomes.

Finally, the study did not collect in-depth data on intervention usage, which means the study is limited in being able to talk to the fidelity of the training or its short- or long-term use by the participants. This will be an important focus area for future studies on the Be Well Plan.

To conclude, this study provides evidence for the impact of an internet-based mental health intervention during a period of significant community need. The intervention resulted in improvements in both participant mental well-being and psychological distress. After analyzing within-individual effects of the program, a differential response pattern was observed, indicating that improvement in mental well-being and reduction in psychological distress were not necessarily congruent. This indicates the importance of assessing dimensions of both well-being and distress when determining intervention effectiveness, which in the case of the Be Well Plan, added evidence to the impact that internet-based mental health promotion interventions can have generally and during times of societal distress such as pandemics.

## Abbreviations

ACT: Acceptance and Commitment Therapy
DASS: Depression Anxiety and Stress Scale
MANOVA: multivariate analysis of variance
MHC-SF: Mental Health Continuum Short-Form
RCI: reliable change index
SAHMRI: South Australian Health and Medical Research Institute
SWLS: Satisfaction With Life Scale
